# Cytokine-Targeted Therapeutics for KSHV-Associated Disease

**DOI:** 10.3390/v12101097

**Published:** 2020-09-28

**Authors:** Nedaa Alomari, Jennifer Totonchy

**Affiliations:** Department of Biomedical and Pharmaceutical Sciences, Chapman University School of Pharmacy, Irvine, CA 92618, USA; aloma112@mail.chapman.edu

**Keywords:** KSHV, pathogenesis, cytokine signaling, cytokine therapeutics, cytokine targeted therapy, immunomodulatory therapeutics

## Abstract

Kaposi’s sarcoma-associated herpesvirus (KSHV) also known as human herpesvirus 8 (HHV-8), is linked to several human malignancies including Kaposi sarcoma (KS), primary effusion lymphoma (PEL), multicentric Castleman’s disease (MCD) and recently KSHV inflammatory cytokine syndrome (KICS). As with other diseases that have a significant inflammatory component, current therapy for KSHV-associated disease is associated with significant off-target effects. However, recent advances in our understanding of the pathogenesis of KSHV have produced new insight into the use of cytokines as potential therapeutic targets. Better understanding of the role of cytokines during KSHV infection and tumorigenesis may lead to new preventive or therapeutic strategies to limit KSHV spread and improve clinical outcomes. The cytokines that appear to be promising candidates as KSHV antiviral therapies include interleukins 6, 10, and 12 as well as interferons and tumor necrosis factor-family cytokines. This review explores our current understanding of the roles that cytokines play in promoting KSHV infection and tumorigenesis, and summarizes the current use of cytokines as therapeutic targets in KSHV-associated diseases.

## 1. Introduction

In 1994, Kaposi’s sarcoma-associated herpesvirus (KSHV) was first identified in a Kaposi’s sarcoma (KS) lesion by Chang and Moore [1]. KS is a highly proliferative tumor derived from lymphatic endothelial cells [2]. KSHV infection is also linked to two B cell lymphoproliferative disorders—primary effusion lymphoma (PEL) and multicentric Castleman’s disease (MCD) [3,4]. In addition, recent studies have demonstrated a third KSHV-associated disease. KSHV-associated inflammatory cytokine syndrome (KICS) is characterized by high levels of viral interleukin-6 (vIL6) or human IL-6 (hIL-6) [5]. Cytokine production has been shown to contribute to KS pathogenesis [6,7,8]. In fact, high levels of IL-6, TNF-α and IL-10 were identified in sera of KS patients [9] and KS tumors have shown elevated level of IL-6, IL-10 and IFN-γ [10]. Furthermore, in vitro studies have reported that high production of proinflammatory cytokines, such as IL-6, TNF-α, CCL-2 and, CXCL-10, may contribute to the progression of KS [11,12,13,14,15,16,17,18]. One of the mechanisms that may trigger KSHV reactivation, is the secretion of inflammatory cytokines, through activation of Toll-like receptors 7 and 8 (TLR7/8), oncostatin M (OSM), hepatocyte growth factor (HGF) and secretion of interferon-γ (IFN-γ) when stimulated by co-infection with other viruses [19,20]. Previous reports have also shown that KSHV infection induces proinflammatory cytokines including IL-1a, IL-1b, which are implicated in the pathogenesis of KSHV-associated malignancies [21,22].

Cytokines are soluble messengers controlling immune responses, and are involved in myriad biological process including inflammation, cell proliferation and cell migration. Manipulation of cytokines may have therapeutic benefit in diseases ranging from cancer to autoimmune manifestations and infectious disease. Multiple recombinant cytokines have been approved by the Food and Drug Administration (FDA) including IL-2 for the treatment of advanced renal cell carcinoma (RCC) [23] and IFN-α for the treatment of hairy cell leukemia [24] and Acquired immunodeficiency syndrome (AIDS)-related Kaposi’s sarcoma [25]. Development of cytokine-based therapies that target immunomodulatory mechanisms may be promising treatments for KSHV-associated diseases. Indeed, tocilizumab, the first humanized IL-6 receptor blocking antibody, has been approved for treatment of MCD in Japan. As knowledge of KSHV-associated diseases has grown, the research and the clinical trials have expanded to include cytokine targeted therapeutics (Table 1).

## 2. IL-6

Interleukin-6 is an inflammatory cytokine with pleiotropic effects produced by many cells in the body, such as epithelial cells, muscle cells, hematopoietic cells and stromal cells. It plays a role in various biological processes, including cancers and immune system diseases [26,27]. In the early stages of inflammation, monocytes, and macrophages secret IL-6 in response to activation of Toll-like receptors. Upregulation and persistent IL-6 stimulation are observed in in several inflammatory and autoimmune diseases [28]. The KSHV K2 gene [29] encodes a protein, viral IL-6 (vIL-6), that shares 24.8% amino acid similarity with human interleukin-6 (hIL-6) [29,30]. Several studies have uncovered roles of vIL-6 in KSHV-associated diseases, reviewed in [31]. Recently, a new in silico study presented the significant similarity between vIL6 and hIL6 folding and used this analysis to describe the similarity of the functions or interactions of both proteins, and defined four amino acid to be a new target to inhibit the pathological role of vIL6 [32]. A recent pilot study in 25 children with human immunodeficiency viruses (HIV)-associated KS in Malawi has demonstrated associations between KSHV viral load and hIL-6 levels in the blood, which may contribute to disease progression. [33]. Expression of vIL-6 protein has been detected in serum from patients with KS, PEL and MCD [34], and high levels of plasma hIL-6 drive progression of MCD and correlate with poor prognosis in PEL [35]. Moreover, experiments performed in AIDS/KS-derived cells demonstrated that hIL-6 is required for tumor cell proliferation [11]. vIL-6 can also serve as autocrine growth factor for PEL cell lines [36], and induces vascular endothelial growth factor (VEGF) in vitro and in vivo which plays a major role in PEL pathogenesis [37]. In addition, a study has reported that Tocilizumab, inhibited ascites formation in the BCBL-1 intraperitoneal xenograft mouse model of PEL [38], suggesting that hIL-6 is a viable therapeutic target in PEL. Patients with KICS, show elevated levels of hIL- 6 and vIL-6 [5]. KICS is one of the most aggressive clinical manifestations linked to KSHV infection, and it is associated with cytokine storm including high levels of hIL-6 [5], due to the overproduction of hIL-6 with high KSHV viral load it is most likely to contribute to the clinical features of the disease. Moreover, there is significant crosstalk between vIL-6 and hIL-6. vIL-6 can induce endogenous hIL-6 secretion in patients with MCD [39]. In an MCD mouse model, vIL-6 transgenic mice that have serum levels of vIL-6 comparable to KSHV-infected patients only develop the MCD-like phenotype if endogenous IL-6 is also present [40]. In 23 HIV/MCD patients, a strong correlation was found between the HHV8 viral load and plasma hIL-6 [41]. Due to the contribution of hIL-6 in the biology of KSHV and the elevated serum levels of hIL-6 which may contribute to the clinical features of MCD, inhibiting the activity of hIL6 is a promising therapeutic strategy for MCD which already has some data to support it. Current anti-IL-6 therapeutics include tocilizumab, an anti–interleukin-6 receptor antibody, and siltuximab, anti–hIL-6 monoclonal antibody. Both of these therapeutics are currently being used in clinical trials for therapeutic efficacy in MCD, and have shown promise when used either preventively or therapeutically [41]. One study has reported the efficacy of a rat anti-mouse IL-6R antibody, MR16-1, in a murine model of MCD in which transgenic mice carry a human IL-6 cDNA fused with a murine major histocompatibility class-I promoter (H-2Ld). Treatment of these mice with MR16-1 improved most of the MCD-like symptoms in compared with controls [42]. In humans, a clinical study evaluating the safety and efficacy of tocilizumab in 28 MCD patients for 16 weeks, showed that tocilizumab therapy alleviated the inflammatory symptoms and biochemical abnormalities associated with MCD with sustained improvement over one year [43]. Another retrospective study has reported that eleven out of twelve MCD patents treated with tocilizumab had partial remission and three achieved complete remission [44]. An additional Phase II clinical trial with tocilizumab, is currently in progress to determine if it will be an effective therapy for KSHV-MCD. (NCT01441063). In this study, 18 out of 23 patients (78%) had clinical benefits response and 12 patients (52%) showed objective tumor response. All 11 patients treated with the highest dose of 12 mg/kg had clinical benefit response and eight patients (73%) showed objective tumor response. A recent open label, single center pilot study has evaluated tocilizumab (8 mg/kg) administered intravenously on Day One of 14-day cycles for a maximum of six cycles both alone and in combination with zidovudine (AZT) and valganciclovir (VGC) in 8 KSHV-MCD patients with HIV. All patients in the study were on antiretroviral therapy (ART). One patient had complete clinical remission and four patients had partial responses on tocilizumab alone within the first two cycles of treatment. Of three patients who had AZT/VGC in combination with tocilizumab, two had partial responses and one had a complete remission response within four weeks of treatment [45]. The initial data for siltuximab activity comes from a Phase I, open-label, dose-finding study reported by Van Rhee and colleagues in 23 patients with symptomatic, multicentric or unresectable unicentric Castleman disease [46]. Moreover, a randomized, double-blind, placebo-controlled trial in 79 MCD patients treated with siltuximab showed a major benefit, 18 (34%) of 53 patients in the siltuximab group had durable tumor and symptomatic responses while no response was seen in 26 patients in the placebo group [47]. Further studies and clinical research are needed to better understand the role of elevated hIL-6 in MCD in order to develop this paradigm into an effective treatment strategy.

## 3. IL-10

Interleukin 10 is a potent anti-inflammatory cytokine produced by several immune cells, including dendritic cells, macrophages, B and T cells. Originally, IL-10 was labeled as cytokine synthesis inhibitory factor (CSIF) due to its activity as an inhibitor factor to the production of proinflammatory IFN-γ and TNFα cytokines by T helper l (Th1) cells. IL-10 has critical roles in the immune system primarily associated with regulating and suppressing the induction of proinflammatory cytokines to protect the host from inflammatory tissue damage. [48,49]. Several herpesviruses including cytomegalovirus (CMV), equine herpesvirus type 2 (EHV-2) and Epstein-Barr virus (EBV) encode viral homologs of IL-10, which contribute to immunosuppressive activity and suppress inflammatory responses [50,51,52]. The viral IL-10 homologs show immunomodulatory activities such as inhibition of DC maturation in both infected and bystander cells [53]. Many studies have suggested that anti-inflammatory cytokines including IL-10, are associated with the KSHV infection and/or AIDS KS development [54,55,56]. KSHV replication and transcription activator protein (K-RTA), activates the promoter of the human *IL-10* gene [57]. K-RTA is an immediate early gene and is critical for the induction of lytic replication and the spread of KSHV infection [58,59,60]. Interestingly, studies have reported the induction of IL-10 secretion by macrophages during the KSHV infection or expression of the viral miRNAs miR-K12-3 and miR-K12-7 through targeting C/EBPβ p20 (LIP), a negative transcription regulator of IL-10 [61]. STAT3 activation resulting from dendritic cells exposure to UV-inactivated KSHV was correlated to IL-10 production [61]. More recently, studies have shown an elevated level of serum IL-10 in visceral AIDS-KS patients compared to HIV-positive individuals and classic KS patients [62]. In addition, IL-10 is expressed by PEL cells in vitro and in vivo and serves as autocrine growth factor for PEL-derived BCBL-1 and BC-1 cells [36]. Similarly, studies have demonstrated the involvement of elevated levels of cellular cytokines including IL-10 in the pathogenesis of KSHV-associated diseases, including PEL and MCD in patients with HIV/AIDS [36,54,63,64]. These studies suggest that IL-10 may have an important role in the development and progression of KSHV-associated diseases. A recent case study reported an elevated level of IL-10 and KSHV load in a KICS patient who, remarkably, not have elevated levels of hIL-6 [65]. These data indicate the powerful role of IL-10 in the pathogenesis of KSHV and the potential benefit of IL-10 as a biomarker for KICS patients. Neutralization of IL-10 been studied as therapeutic approach in viral infection [49,66]. Notably, IL-10 blockade restores IFN-γ production on HIV-1-Specific CD4 T Cell [67] and restores function of HIV specific and HCV specific T cells in vitro [68,69]. Thus, manipulation of IL-10 may hold promise as an immunotherapeutic strategy for KSHV-associated disease.

## 4. Interferons

Interferons (IFNs) are cytokines that play an important role in the immune system with potent antiviral activity. IFNs have a critical defense role against intracellular pathogens. In general, IFNs are classified into three types, Type I, Type II and Type III. Type I IFNs include IFN-α and IFN-β, which are released by virus infected cells. Type II IFNs include IFN-γ, which is secreted by natural killer cells, CD4+ T helper (Th1) cells and CD8+ cytotoxic T cells [70,71]. Although each type of IFN signals through different receptors, there is overlap in the signal transducing responses such as Jak-STAT signaling. [72,73,74,75]. While all types of IFNs induce the expression of antiviral proteins, there are divergent responses in KS patients. Patients who showed a response to IFN-α treatment with regression of KS lesions had an adequate number of CD4+ T cells compared to non-responders. [76,77,78]. KSHV infection upregulates the TLR3 pathway and its downstream target, interferon β (IFN-β) in monocytes [15]. In addition, KSHV infection induces IFN-α expression through TLR9 signaling in plasmacytoid dendritic cells [79]. Many KSHV proteins target the interferon response, and this inhibition may contribute to KSHV pathogenesis. Immediate-early lytic genes RTA and ORF45 inhibit the type I IFN pathway [80,81,82]. vIRFs are known to interact with cellular IRFs to block IFN production (reviewed in [83]). IRFs are best known for their rule in the regulation of IFN production and inflammatory responses downstream of pattern recognition receptors [84]. The latency-associated protein of KSHV, LANA inhibits the interferon pathway through binding to the IFN-β promoter [85]. Clinical trials for the treatment of KS patients with IFN-γ were halted due to unforeseen progression of KS lesions [86,87]. However, several groups have reported that viral lytic gene expression in BCBL-1 or BC-1 cells is promoted by IFN-γ [19,88]. Interestingly, recent data have shown inhibitory effects in viral progeny production with the IFN-γ treatment in infected primary human lymphatic endothelial cells (LECs) as well as induced KSHV-producer cells (iSLK.219) [89]. Moreover, treatment of BCBL-1 cells with IFN-γ induced expression of antiviral proteins such as double-stranded RNA-activated protein kinase (PKR) [90]. A recent study has reported the association between interferon lambda (IFNL3/4) polymorphisms and susceptibility to KS in HIV-infected individuals among men who have sex with men [91]. To date, there are only two interferon therapeutics that have been approved for KSHV-associated diseases, recombinant IFN-α2a, and IFN- α2b. Complete or partial anti-tumor response were observed in 38% of AIDS-KS patients with treatment of recombinant IFN-α with anti-viral effects in patients with the highest CD4 counts [92]. When low dose of IFN-α with didanosine was used in AIDS-KS, there was a 40% tumor response rate and the median response duration was 110 weeks [93]. On the other hand, an animal model study has demonstrated that IFN-α, in combination with azidothymidine results in significantly increased mean survival time in KSHV infected PEL- engrafted non-obese diabetic/severe combined immunodeficient (NOD/SCID) mice as well as induction of apoptosis in PEL cells [94]. Due to its side effects, IFN-α is rarely used therapeutically. However, the pegylated formulations of IFN-α showed partial or complete tumor response in 8 of 10 AIDS-KS patients, all were on combination antiretroviral therapy (cART) at peg-IFN initiation [95]. Another case has reported that pegylated formulations of IFN-α were efficient against KS skin lesions in AIDS-KS patient who were on cART [96]. Taken together, this evidence suggests that combining pegylated IFN-α with antiviral therapy may display potent efficacy against KSHV-associated diseases.

## 5. TNFa

Tumor necrosis factor (TNF) plays a major role in host defense mechanisms and the immune response [97]. TNF cytokines were described based on their ability to kill tumor cells in vitro and to cause hemorrhagic necrosis of transplantable tumors in mice [98,99]. The TNF family includes TNF-α and TNF-β. The role of TNF-α in KSHV infection is complex. In vivo studies have correlated TNF-α levels to KSHV infection and the progression of KS [100]. In fact, KSHV stimulates TNF-α production, possibly creating an environment that favors KS disease [101]. Moreover, KS lesions contain elevated levels of TNF-α [102]. A recent study has reported that KSHV glycoprotein B promotes cell adhesion and inhibits cell migration through upregulating TNF-α [103]. On the other hand, another study has reported that TNF-α impaired the production of KSHV virions by 90%, while TNF-β showed moderate inhibition of lytic reactivation in induced iSLK.219 and infected LECs [89]. Elevated levels of TNF-α and NFκB are present in CD19 + cells and serum samples from HIV/AIDS, KSHV coinfected patients who have antibodies against lytic antigens [104]. NF-κB is constitutively activated in KSHV-infected PEL cell lines and activity NF-κB is essential for KSHV-infected lymphoma cells survival [105]. These results suggest that pharmacologic inhibition of NF-κB may be an effective treatment for KSHV associated diseases. The NFκB is a family of transcription factors that control cell survival and may be activated by the recognition of pathogen-associated molecular patterns (PAMPs), such as dsRNA, viral dsDNA and TNF-α. [106]. Activated NFκB regulates the production of a variety of genes involved in immune responses and inflammation signaling, such as TNF-α, IL-1β, IL-2, IL-6, IL-18, IL-12, MCP-1 [107]. Mutation or inappropriate activation of NFκB signaling pathways has been correlated with autoimmunity, chronic inflammation, and cancer [108,109,110].

Furthermore, many studies support an association between TNF blockade and infection or reactivation of KSHV. High incidence of lymphoproliferative disorders has been linked with therapeutic TNF-α blockade [111]. There are many cases published in the literature of KS following treatment with anti-TNF-α including infliximab and golimumab: a case of a rheumatoid arthritis patient who developed KS lesion after infliximab treatment [112] and a patient with giant cell arteritis who developed KS in double-blind trial with anti-TNF-α [113], and a case of psoriatic arthritis patients who developed KS lesions during the course of treatment with infliximab [114]. Further, there was a case with history of psoriasis and psoriatic arthritis who developed KS during the treatment with golimumab [115]. However, another example has reported absence of KSHV reactivation after infliximab therapy in rheumatoid arthritis patients [116]. In addition, thalidomide appeared to inhibit TNF-α production [117]. A recent study has reported the role of pomalidomide, a thalidomide-like drug, as an immunomodulator with antitumor activity via PD-L1 inhibition [118] and through the restoration of ICAM-1 and B7-2 during latent infection and prevention of down-regulation of MHC-I during lytic activation in PEL cells [119]. Phase II dose-escalation study has reported that thalidomide (100 mg/day for 12 months) improved the clinical manifestation in AIDS-KS, eight out of seventeen patients achieved partial response and two patients had stable conditions [120]. A phase I/II study has evaluated pomalidomide (5 mg/day for 21/28 days), a small molecule derivative of thalidomide, in 22 KS patients with and without HIV infection [121]. 16 patients achieved objective tumor response, 9 out of 15 HIV infected patients achieved objective response, and all of 7 HIV uninfected patients achieved objective response, with median progression-free survival of 16.6 months. Moreover, 10 of 17 patients with edema (59%) showed a decrease in tumor associated edema. Whereas eight patients showed 2-cm reduction of limb circumference and two patients showed substantial subjective improvement. Interestingly, on May 14, 2020, the FDA granted accelerated approval to pomalidomide and expanded the indication to include AIDS-KS and KS patients who are HIV negative. Further studies are needed to address the benefits of anti TNF-α agents alone or in combination and investigate the precise role in other KSHV-associated diseases.

## 6. IL-12

Interleukin 12 (IL-12) was initially described as a “cytotoxic lymphocyte maturation factor” from PMA-induced EBV-transformed B-cell lines. It is a pleiotropic cytokine that links between innate and adaptive immunity [122]. In addition, IL-12 stimulates the production of IFN-γ with anticancer activity [123]. IL-12 has also been implicated in KS pathogenesis. It has been shown that IL-12 down regulates ORF74, the constitutively active G protein-coupled receptor that is encoded by KSHV [124,125,126,127,128]. Thus, IL-12 seemed a good candidate to be used as immunotherapy in humans. However, a very narrow therapeutic index and side effects limited the therapeutic use of this cytokine. Despite those setbacks, IL-12 shows promising results in some cases. Little et al. used IL-12 in a phase 1 pilot study in 32 AIDS-KS patients who were on highly active antiretroviral therapy (HAART), of 24 patients who were treated with higher doses (300, 500, 625 ng/kg), 17 had a complete or partial KS tumor response (61%) [129]. IL-12 was used in a Phase II trial for the treatment of AIDS-KS patients receiving cART, in combination with pegylated liposomal doxorubicin for six, three weeks cycles, followed by (500 ng/kg) IL-12 for 36 weeks, 83.3% had complete or partial KS tumor response [130]. At present, there is an ongoing Phase I/II trial of NHS-IL12, an IL-12 immunocytokine, genetically engineered and composed of two IL-12 heterodimers fused to the NHS76 antibody which targets the therapeutic to necrotic cells, as both a monotherapy and in combination therapy in advanced KS (NCT04303117) [131]. These studies suggest that IL-12 can have potent anti-KS effects in the context of combination therapy. Randomized trials will be needed to further evaluate IL-12 activity in KSHV-associated disease.

## 7. Host and Viral Chemokines

The KSHV genome encodes for three chemokine homologs, vCCL1/vMIP-I, vCCL2/vMIP-II and vCCL3/vMIP-III, encoded by ORFs K6, K4, and K4.1 respectively [29,30,132,133]. All of the chemokines are expressed during the lytic cycle [134,135]. vCCL1 is most highly related to CCL18 (50% amino acid identity) and to human CCL3 (37.9% amino acid identity) [29]. vCCL1 was identified reproducibly as selective viral CCR8 agonist in three independent studies, [136,137,138], suggesting it may function to promote the migration of Th2 lymphocytes, monocytes and endothelial cells. Indeed, vCCL1 is a potent chemoattractant for CCR8+ vascular endothelial cells [139], which could promote dissemination of KSHV within endothelial cells as well as KS tumorigenesis. In addition, vCCL1 was found to play an antiapoptotic role in PEL cells via induction of VEGF-A [140]. vCCL2 binds to a broad spectrum of chemokine receptors, and has primarily inhibitory effects [141]. It can bind to chemokine receptors from all four structural subgroups. In particular, it interacts with CCR1, CCR2, CCR3, CCR5, CCR8, CCR10, CXCR4, CX3CR1, and XCR1 [138,142,143]. vCCL2 blocks the binding of host ligands to CX3CR1 and CCR5, thereby inhibiting the migration of naïve and activated NK cells [144]. In addition, vCCL1 and vCCL2 enhance the survival of endothelial cells and to promote virus productive replication through CCR8 signaling [145]. However, contradicting studies have reported that vCCL2 act as an agonist or as antagonist in different models [146,147,148]. Despite this controversy, vCCL2 may has potential clinical applications. For example, recombinant vCCL2 has been used in rat models of inflammation and it has promising therapeutic effects [146,149]. Moreover, it has potential activity to enhance graft survival after organ transplantation [150,151,152], and a synthetic peptide derived from the N-terminus of vCCL2 can inhibit HIV-1 replication in CXCR4+ T-cell lines [153,154]. vCCL3 is related to vCCL1 and vCCL2 (25–28% amino acid identity) and most highly homologous to human XCL1. vCCL3 is highly selective and potent agonist at the human lymphotactin receptor XCR1, suggesting that it functions in KSHV immune evasion by modifying the migration and function of antigen presenting cells [138]. Furthermore, a study has reported that vCCL3 is CCR4 agonist [155]. This finding also supports the characterization of vCCL3 as an immunomodulatory factor, suggesting that it may modulate the function of regulatory T cells and antigen presenting cells during infection.

KSHV also encodes a chemokine receptor homolog, called ORF74. ORF74 has 27% homology with CXCR2 which are expressed in KS lesions [124]. ORF74 displays a broad chemokine-binding repertoire; binding to CXCR2 ligands including CXCL1, CXCL2, CXCL3, CXCL5, CXCL6, CXCL7, CXCL8 and CC chemokines CCL1 and CCL5 [156,157]. Remarkably, it has been reported that ORF74 is constitutively active and induces a variety of intracellular signaling pathways [157]. CXCR2 ligands generally function as ORF74 agonists [156,158], while non-CXCR2 ligands including CXCL10 and CXCL12 act as inverse agonists [125,156,159]. Studies have reported KSHV infection of human monocytes upregulates human CCL2, and CXCL10 transcripts [15], and production of CXCL1 and CCL5 in SiHa cell [160]. CCL2 is correlated with increased angiogenesis and migration of tumor cells, which may drive KS progression, and immune cell migration [161,162]. In endothelial cell models, it has been reported that KSHV promotes the production of CCL2 via induction of ATF4 [163]. Moreover, vIL-6 signaling in endothelial cells increases CCL2 secretion via induction of hypoxia-upregulated protein 1 [164]. Furthermore, CXCL12/CXCR4 and CXCR7 are upregulated in KS and correlates with the severity of KS lesions [165].

CXCL1 has been investigated as a therapeutic target and potential diagnostic biomarker in other cancers [166,167]. A recent study has tested an anti-CXCL1 monoclonal antibody to inhibit the growth of bladder and prostate tumors [168]. In addition, CCL2-targeted therapy has been studied intensively in the field of cancer, especially in combination therapies [169,170,171]. These findings indicate that chemokine-targeted therapeutics may represent useful therapeutic targets in viral cancers, and highlight the potential role of chemokines as biomarkers for KSHV diagnosis and progression, but further information is needed to understand the nuance of chemokine dysregulation in KSHV disease before it can be exploited as a therapeutic paradigm.

## 8. Summary and Future Considerations

**Figure 1:**
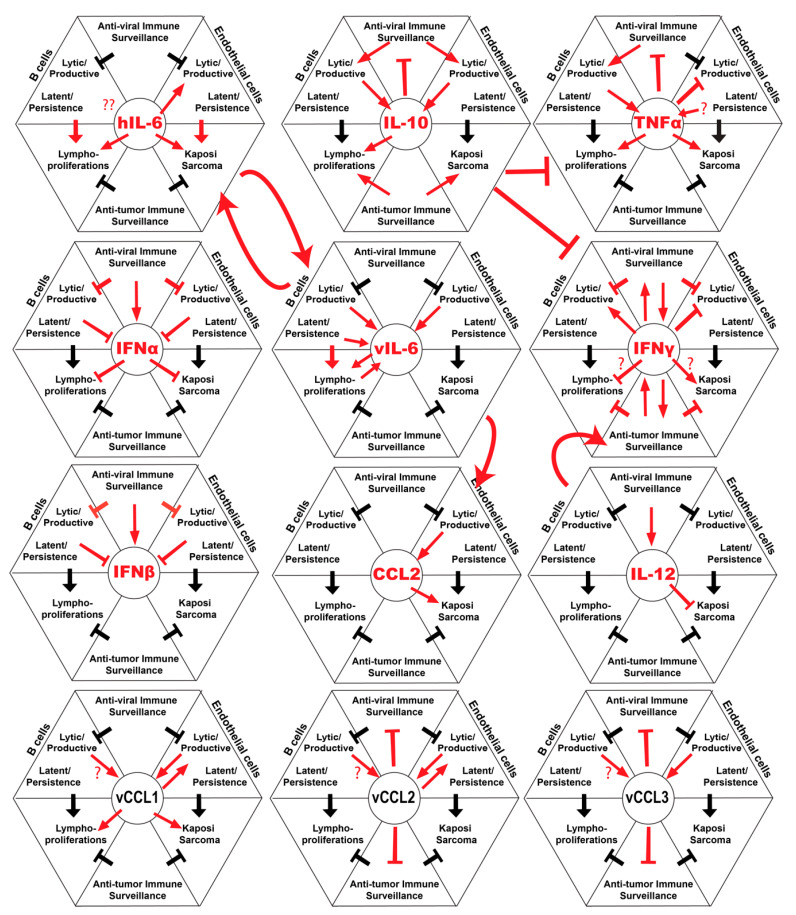
Summary of host and viral cytokines and their interactions with Kaposi’s sarcoma-associated herpesvirus (KSHV) infection, immunity and tumor progression.

Cytokine based therapies have great potential for treating a variety of diseases including cancers and infectious diseases. Our understanding of the complex roles of cytokines in KSHV infection and disease is advancing, but many questions remain. In this review, we have attempted to summarize the current state of knowledge in this niche and highlight areas where cytokines may represent therapeutic targets for KSHV-associated diseases (Figure 1). Clearly, understanding the role of the cytokines during the immune response and their mechanisms in the biology of KSHV infection and tumorigenesis will facilitate development of new targets and enhance the efficacy of exiting cytokine-targeted therapies. The success of specific cytokine-targeted therapeutics such as IL-6, IL-15, IL-2 and IFN-α in different malignancies, will give rise to novel strategies to consider in the future of KSHV therapy. In addition, many diseases KSHV have similar clinical presentations and are difficult to diagnose histologically, therefore cytokine profiles may be considered to be a useful tool as biomarkers to differentiate these conditions. Genetic association studies of IFN lambda polymorphisms in KS, suggest a potential role of this cytokine in disease, and it will be interesting to determine if this class of interferons has therapeutic potential in KSHV-associated malignancy. Current research in the field of immunobiology of cytokines has resulted in promising ongoing clinical trials for cytokine-targeted therapy in KSHV-associated disease. Continued research in this field will provide additional insights into the major role of cytokines in the biology and pathogenesis of KSHV infection and KSHV-related diseases.

## Figures and Tables

**Table 1:** Summary of the clinical trials referenced in this review.

Cytokine	KSHV Disease	Drug	Dose	Study Type	Summary of the Result
IL-6	MCD	Tocilizumab	8 mg/kg MRA biweekly	Open-label trial in 28 patients for 16 weeks [43]	Alleviated the inflammatory symptoms and biochemical abnormalities
			8 mg/kg every 2 weeks	Ongoing Phase II clinical trial (NCT01441063).	
		Tocilizumab Zidovudine Valganciclovir	8 mg/kg IV on day 1 of 14-day cycles for a maximum of 6 cycles 600mg every 6 h 900mg every 12 h	Open label, single center pilot study [45]	One patient had complete clinical benefit response and four patients had partial clinical benefit responses on tocilizumab alone. Three patients who had AZT/VGC in combination with tocilizumab, two had partial clinical benefit responses and one had complete clinical benefit response
		Siltuximab	Cohorts 1 to 5 enrolled in sequential order. 2-h infusion at 3 mg/kg every 2 weeks, 6 mg/kg every 2 weeks, 12 mg/kg every 3 weeks, 6 mg/kg weekly, and 12 mg/kg every 2 weeks, respectively. Cohort 6, 1-h infusion at 12 mg/kg every 3 weeks. Cohort 7, 9 mg/kg every 3 weeks.	Phase I, open-label, dose-finding and seven-cohort study in 23 patients [46]	18 out of 23 patients had clinical benefits response and 12 patients showed objective tumor response. 11 patients treated with the highest dose of 12 mg/kg had clinical benefit response and 8 patients showed objective tumor response
			11 mg/kg intravenous infusion every 3 weeks	Randomized, double-blind, placebo-controlled trial in 79 patients [47].	18 of 53 had tumor and symptomatic responses
IFNa	AIDS-KS	IFNa	Starting dose 35×10^6^ Reduced to 5–10×10^6^	Open therapeutic trial [92]	Anti-viral effects Anti-tumor response in 38% of patients
	AIDS-KS	IFNa with didanosine	1 million (low dose) or 10 million (intermediate dose) IU per day by subcutaneous (s.c.) injection.	randomized phase II clinical trials [93]	40% tumor response
TNFa	AIDS-KS	Thalidomide	100 mg/day for 12 months	Phase II dose-escalation study [120]	Improved the clinical manifestation 8 out of 17 patients achieved partial response and 2 patients had stable conditions
	AIDS-KS and KS	Pomalidomide	5 mg/day for 21/28 days	phase I/II study in 22 KS patients with and without HIV infection. [121]	16 patients. Achieved objective tumor response and 9 out of 15 HIV infected patients. Achieved objective response.
IL-12	AIDS-KS	L12	100, 300, 500 and 625 ng/kg	Phase 1 pilot study [129]	17 had a complete or partial KS tumor response (61%) with three highest does.
	AIDS-KS	IL12 alone and in combination with pegylated liposomal	300 ng/kg subcutaneously twice weekly for six 3-week cycles, followed by 500 ng/kg subcutaneous IL-12 twice weekly	Phase II clinical trial [130]	83.3% complete or partial KS tumor response.
	Advanced KS	NHS-IL12		Ongoing Phase I/II clinical trial (NCT04303117)	
